# Notch-1 Signaling Modulates Macrophage Polarization and Immune Defense against *Mycobacterium avium paratuberculosis* Infection in Inflammatory Diseases

**DOI:** 10.3390/microorganisms8071006

**Published:** 2020-07-05

**Authors:** Esra’a Keewan, Saleh A. Naser

**Affiliations:** Division of Molecular Microbiology, Burnett School of Biomedical Sciences, College of Medicine, University of Central Florida, Orlando, FL 32816, USA; Esraakeewan@knights.ucf.edu

**Keywords:** notch, macrophages, paratuberculosis, IL-6, mcl-1, apoptosis, polarization, M1, M2, Crohn’s disease, rheumatoid arthritis

## Abstract

Despite the extensive research on Notch signaling involvement in inflammation, its specific role in macrophage response in autoimmune disease and defense mechanisms against bacterial infection, such as *Mycobacterium avium paratuberculosis* (MAP), remains unknown. In this study, we investigated the molecular role of Notch-1 signaling in the macrophage response during MAP infection. In particular, we measured the in vitro effect of MAP on Notch-1 signaling and downstream influence on interleukin (IL)-6 and myeloid cell leukemia sequence-1 (MCL-1) and consequent cellular apoptosis, MAP viability, and macrophage polarization. Overall, the data show significant upregulation in Notch-1, IL-6, and MCL-1 in MAP-infected macrophages, parallel with a decrease in apoptosis and elevated pro-inflammatory response in these infected cells. On the contrary, blocking Notch signaling with γ-secretase inhibitor (DAPT) decreased MAP survival and burden, increased apoptosis, and diminished the pro-inflammatory response. In particular, the treatment of infected macrophages with DAPT shifted macrophage polarization toward M2 anti-inflammatory phenotypic response. The outcome of this study clearly demonstrates the critical role of Notch signaling in macrophage response during infection. We conclude that MAP infection in macrophages activates Notch-1 signaling and downstream influence on IL-6 which hijack MCL-1 dependent inhibition of apoptosis leading to its chronic persistence, and further inflammation. This study supports Notch-1 signaling as a therapeutic target to combat infection in autoimmune diseases such as Crohn’s disease and Rheumatoid Arthritis.

## 1. Introduction

Notch signaling is an evolutionary-conserved pathway that mediates juxtracrine cell communication through the receptor–ligand interaction. Four Notch receptors, known as Notch 1–4, and five ligands, known as Delta-like (DLL) 1, 3, 4 and Jagged 1–2, have been recognized in mammals. The activation of the Notch receptor by the transmembrane ligand on juxtaposed cells leads to proteolytic cleavage of the Notch receptor by the γ-secretase complex, which eventually leads to the release and nuclear translocation of the Notch intracellular domain (NICD), where it interacts with DNA binding protein CSL (C promotor-binding factor 1 (CBF1)/RBP-J in humans, suppressor of hairless in *Drosophila melanogaster*, Lin-12 and Glp-1 (Lag-1) in *Caenorhabditis elegans*), and activates the transcription of Notch target genes such as hairy enhancer of split (HES) and HES with YRPW motif (HEY) [1].

The Notch signaling pathway coordinates numerous cellular processes throughout the body, including immunity [2]. A considerable body of literature has confirmed the role of Notch signaling in immune cell development and function [1,2]. Although the role of Notch signaling in lymphocyte development and functions is well accepted, there is still considerable ambiguity about the role of Notch signaling in myeloid cells including macrophages [3]. This includes the role of Notch signaling in the macrophage immune response, and defense mechanisms against intracellular bacterial infections which remain unknown.

Macrophages are considered the key effector cells of innate immunity that serve as the first line of defense to encounter invading pathogens [4]. However, they are the primary target for infection by various pathogens, including *Mycobacterium avium* subspecies *paratuberculosis* (MAP) [5]. MAP is an obligate intracellular pathogen that has been associated with several pathological conditions such as Crohn’s disease (CD) [6], Type 1 diabetes [7], and Rheumatoid Arthritis (RA) [8]. MAP infects and persistently survives in macrophages by adopting several survival strategies to escape host defense mechanisms [5]. The apoptosis of infected macrophages is considered one of the host defenses to eradicate the infected cell and its resided bacteria [9]. Additionally, apoptotic macrophages present bacterial antigens to the adaptive immune system to ensure bacterial effective clearance and constraint the infection [10]. 

Notch signaling has been reported to regulate apoptosis in various cell types [11]. However, the role of Notch signaling in regulating macrophage apoptosis, particularly during MAP infection, is still poorly determined. Evidence suggests the involvement of Notch-1 signaling in regulating macrophage apoptosis by inducing the expression of myeloid cell leukemia sequence-1 (MCL-1) [12]. MCL-1 is an anti-apoptotic protein that forms heterodimerization with pro-apoptotic protein Bak and Bax to abrogate their apoptotic effect [13]. In macrophages, the expression level of MCL-1 is mediated by several factors including STAT3 [14], a transcription factor that mediates cellular responses to various cytokines including interleukin (IL)-6 [15]. Previously, we reported the upregulation of IL-6 expression and the consequent increase in bacterial viability in MAP-infected macrophages [16]. Several reports have described the regulatory role of Notch-1 on IL-6 expression, as the IL-6 promoter in humans and mice showed a conserved binding motif of Notch signaling protein [17,18]. This hints to a possible involvement of Notch-1 signaling in macrophage immune response and defense mechanisms against MAP infection.

The plasticity of the macrophage allows for the switch of its phenotypic response according to microenvironmental cues. In particular, Macrophages may assume pro-inflammatory phenotype M1 in response to interferon (IFN)-γ, tumor necrosis factor (TNF)-α and bacterial lipopolysaccharide (LPS). On the other hand, IL-4 and IL-13 promote macrophages to adopt M2 anti-inflammatory phenotype [19]. Therefore, the proper activation of the macrophage is important to achieve the successful elimination of the invading pathogens or an insulting stimulation, while a disparity in macrophage activation is associated with pathological conditions including autoimmune diseases [20,21]. Accordingly, elucidating the signaling pathways that coordinate macrophage activation under different conditions is essential for understanding the molecular basis involved in the development of diseases, including MAP-associated disorders. The role of Notch-1 signaling in macrophage polarization in response to MAP infection has not previously been reported. In this study, we investigated the in vitro effect of MAP infection on Notch-1 signaling and downstream influence on IL-6 and MCL-1 expression in infected macrophages. In particular, we evaluated the role of Notch-1 signaling in apoptosis and consequent MAP viability and its effect on macrophage polarization and inflammatory response in MAP-infected macrophages.

## 2. Materials and Methods

### 2.1. Cell Culture and Treatment

The human monocytic cell line THP-1 (ATCC TIB-202) was used to model macrophages. THP-1 cells were cultured in RPMI-1640 medium (Thermo Fisher, Cat# A1049101, Waltham, MA, USA) containing 10% fetal bovine serum (FBS) (Thermo Fisher, Cat# 26140) and maintained in a humidified 5% CO₂ incubator at 37˚ C. THP-1 monocytes were differentiated to THP-1-derived macrophages with 50 ng/mL of phorbol 12-myristate 13-acetate (PMA) (Sigma-Aldrich, Cat# 8139, Missouri, USA) for 48 h. Cells were then washed with phosphate buffer saline (PBS) (Thermo Fisher, Cat# 20012050) and incubated with culture media for further processing.

To target Notch signaling in THP-1-derived macrophages, the γ-secretase inhibitor DAPT [*N*-(*N*-[3,5-difluorophenacetyl]-l-alanyl)-*S*-phenylglycine *t*-butyl ester] (Cell Signaling Technology, Cat# 15020, Danvers, MA, USA) was used, cells were treated with DAPT (0–40 μM) for 24 h. To inhibit MCL-1 in THP-1-derived macrophages, Maritoclax (MCL-1 inhibitor) (Tocris, Cat# 5368, Minneapolis, MN, USA) was used, cells were treated with Maritoclax (0–80 μM) for 24 h.

### 2.2. Bacterial Culture

The clinical MAP strain (UCF4; isolated from CD patient) and *Mycobacterium smegmatis* (ATCC 27199BD) were cultured using Bactec MGIT Para-TB medium tubes (Becton Dickinson, Cat# 245122, NJ, USA). Bactec MGIT Para-TB supplements (Bovine albumin, Catalase, Casein, and Oleic acid) was added for MAP tubes. Bactec MGIT Para-TB medium tube contains 7 mL of modified Middlebrook 7H9 broth base and an oxygen-sensitive fluorescent sensor embedded on the bottom of each tube, where actively respiring bacteria cause the sensor to fluoresce. All Bactec MGIT Para-TB medium tubes were incubated in BD Bactec^TM^ MGIT^TM^ 320 Analyzer at 37° C.

### 2.3. Quantitative Real-Time PCR (RT-PCR)

Total RNA was isolated from cells using TRIzol™ reagent (Thermo Fisher, Cat# 15596018) according to the manufacturer’s instructions; 800 ng of total RNA was used to synthesize cDNA, and quantitative real-time PCR was performed using the StepOnePlus^TM^ Real-Time PCR System (Thermo Fisher, Cat# 4376600) with Fast SYBR Green Mastermix (Thermo Fisher, Cat# 4385610) as detection dye. Housekeeping β-actin primer (Thermo Fisher) was used to measure the endogenous baseline CT values. Relative mRNA expression levels were calculated by using the equation 2^(−ΔCT)^ × 1000, where ΔCT= [(Sample RT-PCR CT value) − (β-actin CT baseline value)]. The primers (Thermo Fisher) used for the RT-PCR in this study are shown in Table 1.

### 2.4. Measurement of MAP Viability and Load

To measure relative MAP viability in infected macrophages, Live/Dead^TM^ *Bac*light^TM^ bacterial viability assay (Thermo Fisher, Cat# L7007) was used. Briefly, THP-1-derived macrophages were infected with MAP (UCF 4, 10^7^ CFU/mL) for 24 h, after washing to remove extracellular bacilli, cells were collected and lysed with 500 µL of M-PER^™^ Mammalian reagent (Thermo Fisher, Cat# 78503). Samples were centrifuged at 10,000 × *g* for 10 min, then 100 μL of each sample supernatant was loaded in triplicate into separate wells of 96-well flat-bottom microplate and mixed with 100 μL of staining reagent mixture (SYTO^®^ 9 green- fluorescent nucleic acid stain and Propidium iodide red-fluorescent nucleic acid stain). Following 15 min of incubation in the dark, the integrated intensities of the green (530 nm) and red (630 nm) emission and excitation at 485 nm were measured using SpectraMAX^®^ i3x Multi-mode microplate reader. Then the green/red fluorescence ratios were calculated, which detected the Live/Dead MAP ratio. The least-squares fit line was generated from various proportions of Live: Dead MAP, and the equation was used to calculate the MAP viability represented as colony-forming units per milliliter (CFU/mL). The direct effect of DAPT on extracellular MAP growth in Bactec MGIT Para-TB medium tubes was evaluated by BD Bactec^™^ MGIT^™^ 320 Analyzer.

### 2.5. Measurement of Active Cadpase-3 and IL-6 Proteins Levels by Enzyme-linked Immunosorbent Assay (ELISA)

To measure macrophage apoptosis, the level of active caspase-3 cleaved at Asp175/Ser176 in cell lysates was measured using active Caspase-3 ELISA Kit (Thermo Fisher, Cat# KHO1091) following the manufacturer’s instructions. To measure IL-6 secretion level in cell culture medium, IL-6 Human ELISA kit (Thermo Fisher, Cat# BMS213HS) was used following the manufacturer’s instructions. Cleaved caspase-3 and IL-6 levels were determined by reading optical density at 450 nm using SpectraMAX^®^ i3x Multi-mode microplate reader.

### 2.6. Statistical Analysis

To determine the statistical significance in this study, all data were pre-tested for normal distribution using the Kolmogorov–Smirnov normality test followed by Unpaired tow-tailed *t*-test. The values were presented as mean ± standard deviation (SD). All statistical analyses were performed using Prism 8 (GraphPad software, version 8.4.3, San Diego, CA, USA), with *p* < 0.05 considered significant.

## 3. Results

### 3.1. MAP Infection Induces Notch-1, IL-6 and MCL-1 Expression in Macrophage

**Figure 1:**
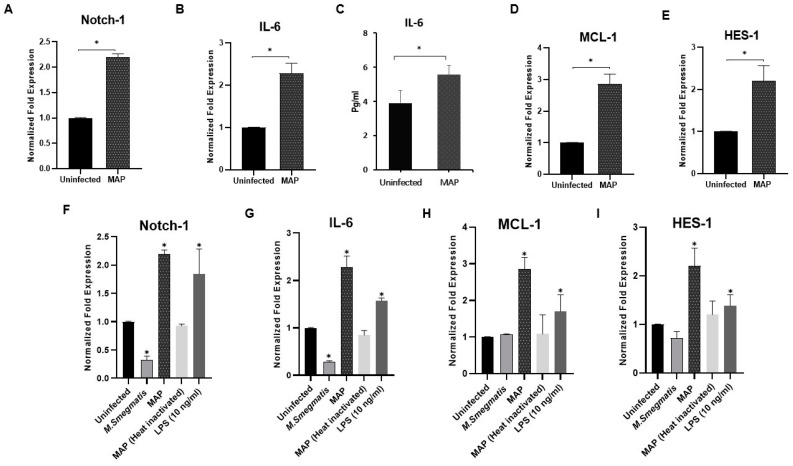
*Mycobacterium avium paratuberculosis* (MAP) induces expression of Notch-1, IL-6, and myeloid cell leukemia sequence-1 (MCL-1) in infected macrophages. (**A**–**E**): Expression level of Notch-1, interleukin (IL)-6, MCL-1, and hairy enhancer of split (HES)-1 was measured in infected macrophages using MAP (UCF 4, 10^7^ CFU/mL) for 24 h. (**F**–**I**): Expression level of Notch-1, IL-6, MCL-1, and HES-1 in macrophages infected with *M. smegmatis,* MAP, heat-inactivated MAP, and lipopolysaccharide (LPS) (10 ng/mL). Gene expression levels were measured using RT-PCR. IL-6 protein level was measured using Enzyme-linked Immunosorbent Assay ELISA. All experiments were performed in triplets. Data are shown as mean ± SD, and significance is considered as * *p* < 0.05.

To explore the effect of MAP infection on Notch-1, IL-6, and MCL-1 expression in infected macrophages, THP-1-derived macrophages were infected with MAP (10^7^ CFU/mL) for 24 h. We found that MAP infection significantly induced Notch-1, IL-6, and MCL-1 gene expression by 1.3, 1.2, and 1.9-fold, respectively, compared to untreated cells (*p* < 0.05; Figure 1A,B,D).

At the protein level, MAP infection significantly induced IL-6 secretion in cell culture medium as shown in Figure 1C (*p* < 0.05).

The ability of MAP infection to induce Notch-1 receptor expression promoted us to determine the impact of MAP infection on Notch signaling activation by measuring the expression of HES-1, a Notch target gene. As shown in Figure 1E, there was a significant upregulation in Hes-1 expression (1.2-fold) in MAP-infected macrophages compared to uninfected cells (*p* < 0.05).

To study the effect of MAP infection compared to the controls on inflammatory response in a macrophage system, Notch-1, IL-6, MCL-1, and HES-1 expression levels were measured in THP-1-derived macrophages. As shown in Figure 1F–I, LPS, which was used as a positive inflammatory control, produced similar effects as observed in MAP infection. In particular, significant upregulation was observed in Notch-1, IL-6, MCL-1, and HES-1 compared to uninfected cells (*p* < 0.05). On the contrary, this effect was not detected in macrophages challenged with heat-inactivated MAP (dead MAP) or *M. smegmatis*, a non-pathogenic related microorganism.

### 3.2. Effect of DAPT on Expression of Notch-1, IL-6 and, MCL-1 in Macrophages

**Figure 2:**
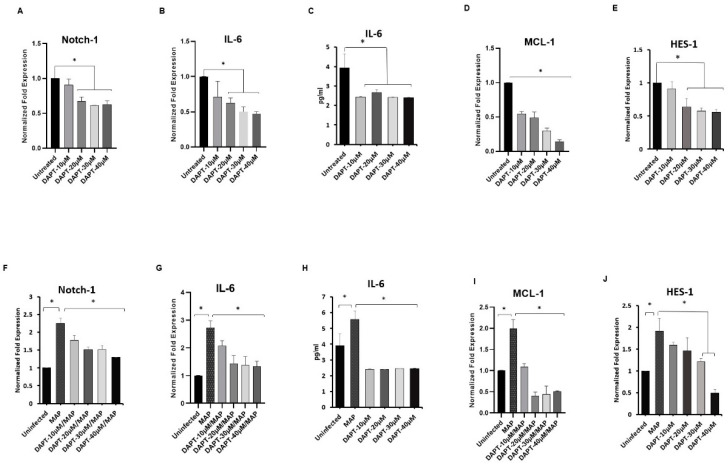
Effect of γ-secretase inhibitor (DAPT) on Notch-1 signaling. (**A–E**): Notch-1, IL-6, MCL-1 and HES-1 expression was measured in macrophages that were pre-treated with DAPT (0–40 μM)**.** (**F–J**): Expression levels of Notch-1, IL-6, MCL-1 and HES-1 in THP-1-derived macrophages that were pre-treated with DAPT then infected with MAP (UCF 4, 10^7^ CFU/mL) for 24 h. The gene expression levels were measured by RT-PCR. IL-6 protein level was measured using ELISA. All experiments were performed in triplets. Data are shown as mean ± SD, and significance is considered as * *p* < 0.05.

To confirm the role of Notch-1 in downstream signaling and its effect on the expression of IL-6 and MCL-1 in THP-1-derived macrophages, Notch signaling was blocked pharmacologically using a γ-secretase inhibitor known as DAPT. In absence of infection, treatment with DAPT (0–40 μM) resulted in a significant decrease in Notch-1 expression at 20–40 μM concentration levels compared to untreated macrophages (average 36%) (*p* < 0.05; Figure 2A). Similarly, IL-6 gene expression decreased significantly at 20–40 μM DAPT concentration compared to untreated macrophages (average 46%) (*p* < 0.05; Figure 2B). IL-6 secretion in cell culture medium was also significantly decreased in response to DAPT treatment by 40% at 10–40 μM concentration levels (*p* < 0.05; Figure 2C). Surprisingly, MCL-1 expression decreased by 45%-86% in response to DAPT treatment at 10–40 μM concentration levels (*p* < 0.05; Figure 2D). To confirm the effect of DAPT on Notch-1 activation, we measured the effect of DAPT treatment on HES-1. We found that the expression levels of HES-1 were significantly reduced by 36%-44% at 20–40 μM DAPT concentration compared to untreated macrophages (*p* < 0.05; Figure 2E). Pre-treatment of THP-1-derived macrophages with DAPT (0–40 μM) for 24 h, followed by MAP infection (10^7^ CFU/mL for 24 h) diminished the ability of MAP to induce Notch-1, IL-6, MCL-1 and HES-1 expression compared with untreated infected group (*p* < 0.05). In particular, 30 μM DAPT pre-treatment resulted in a decrease in the expression of Notch-1 (1.5 ± 0.1), IL-6 (1.4 ± 0.3), MCL-1 (0.44 ± 0.03) and HES-1 (1.2 ± 0.08) compared with the untreated infected group (2.25 ± 0.15, 2.7 ± 0.25, 1.99 ± 0.21, and 1.91 ± 0.29 respectively) (*p* < 0.05; Figure 2F,G,I,J). At the protein level, DAPT (10–40 μM) significantly diminished IL-6 secretion in MAP-infected macrophages by an average of 56% (*p* < 0.05; Figure 2H).

### 3.3. Effect of DAPT on MAP Survival in Macrophage via Notch-1

**Figure 3:**
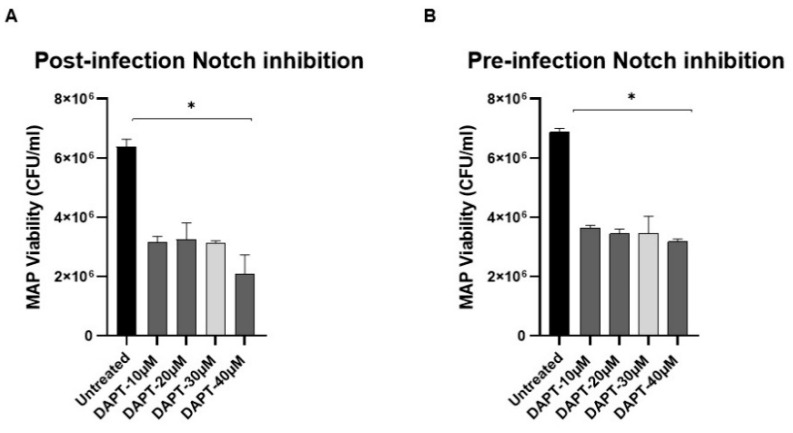
Effect of DAPT on MAP viability. (**A**): MAP viability was measured in THP-1-derived macrophage infected with MAP (UCF 4, 10^7^ CFU/mL) followed by DAPT (0–40 μM) treatment. (**B**): MAP viability was measured in THP-1-derived macrophages pre-treated with DAPT (0–40 μM) followed by MAP infection (UCF 4, 10^7^ CFU/mL)**.** MAP viability was measured using Live/Dead^™^ *Bac*light^™^ bacterial viability assay. All experiments were performed in triplets. Data are shown as mean ± SD, and significance is considered as * *p* < 0.05.

To assess the role of Notch-1 in host defense against infection, MAP viability was measured in DAPT-treated macrophages under two different conditions: Post-infection (Figure 3A) where cells were infected with MAP (10^7^ CFU/mL) for 24 h, then treated with DAPT (0–40 μM) for 24 h, and pre-infection (Figure 3B) where cells were pre-treated with DAPT (0–40 μM) for 24 h, then infected with MAP (10^7^ CFU/mL) for 24 h. To our surprise, DAPT treatment caused a significant decrease (>50%) in MAP viability in both treatment approaches compared to untreated cells (*p* < 0.05). Furthermore, we investigated the direct effect of DAPT on extracellular MAP growth in microbiological medium using Bactec MGIT Para-TB medium treated with DAPT (0–40 μM). There was up to a 45% reduction in MAP growth in DAPT (40 μM)-treated culture groups.

### 3.4. rIL-6 Induces Notch-1, MCL-1 and HES-1 Expression in THP-1-Derived Macrophage

**Figure 4:**
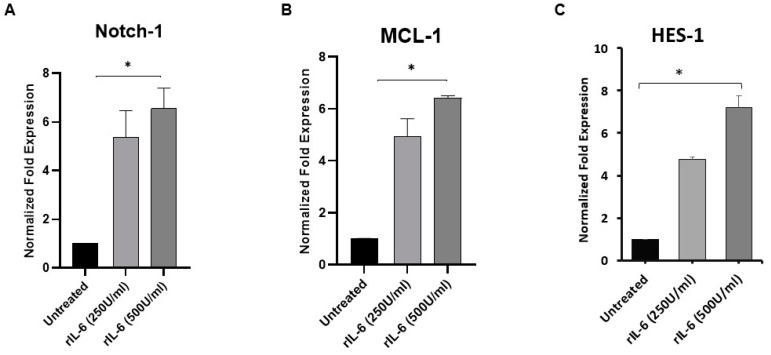
Effect of rIL-6 on Notch-1, MCL-1 and HES-1 expression in macrophages. THP-1-derived macrophages were treated with rIL6 (0–500 U/mL) then analyzed for the expression level of Notch-1 (**A**) MCL-1 (**B**) and HES-1 (**C**) using RT-PCR. All experiments were performed in triplets. Data are shown as mean ± SD, and significance is considered as * *p* < 0.05.

Next, to elucidate the possible interplay between IL-6 and Notch-1 signaling in macrophages, THP-1-derived macrophages were treated with exogenous recombinant rIL-6 (0–500 U/mL) and then analyzed for the expression level of Notch-1, MCL-1 and HES-1. As shown in Figure 4A, rIL-6 treatment caused significant upregulation in Notch-1 expression by 4.3 to 5.5-fold in a dose-dependent manner compared with untreated macrophages (*p* < 0.05), whereas MCL-1 expression was upregulated by 3.9 to 5.4-fold at 250, and 500 U/mL levels, respectively (*p* < 0.05; Figure 4B). Similarly, rIL-6 treatment (250 and 500 U/mL) caused upregulation in HES-1 by 3.8 to 6.2-fold, respectively (*p* < 0.05; Figure 4C).

### 3.5. Effects of rIL-6 on MAP Viability in Infected Macrophages via Notch Signaling

**Figure 5:**
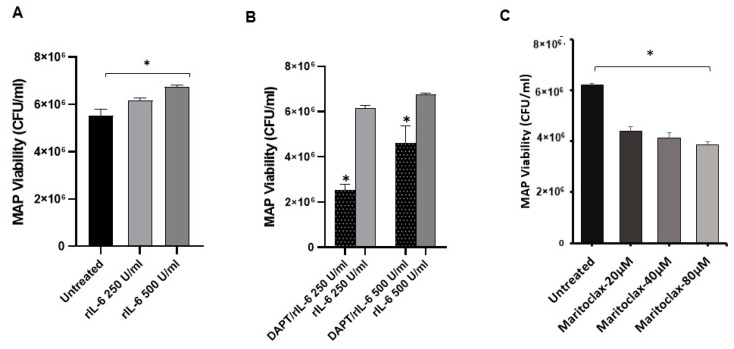
Effect of rIL-6, DAPT and Maritoclax on MAP viability. (**A)**: MAP viability was measured following treatment with rIL-6 (0–500 U/mL) in infected macrophages. (**B)**: MAP viability was measured in infected macrophages which were treated with DAPT (30 μM) then with rIL-6 (0–500 U/mL). (**C**) MAP viability was measured in infected macrophages which were treated with Maritoclax (0-80 μM). MAP viability was measured using Live/Dead^TM^ *Bacl*ight^TM^ bacterial viability assay and represented as (CFU/mL). All experiments were performed in triplets. Data are shown as mean ± SD, and significance is considered as * *p* < 0.05.

To investigate the involvement of Notch signaling in rIL-6-mediated MAP survival in infected macrophages, the effect of rIL-6 on MAP viability in macrophages pre-treated with DAPT (30 μM for 24 h) was examined. As shown in Figure 5A, rIL-6 promoted MAP survival in infected macrophages in a dose-dependent manner (6.2 × 10^6^ – 6.7 × 10^6^ CFU/mL) compared to (5.5 × 10^6^ CFU/mL) in untreated cells (*p* < 0.05). However, DAPT pre-treatment dramatically diminished the ability of rIL-6 to sustain MAP survival in infected macrophages by 31%–59% at different rIL-6 concentration levels (*p* < 0.05; Figure 5B). Interestingly, DAPT pre-treatment caused a decrease in MAP survival in macrophages even in the presence of rIL-6 (2.5 × 10^6^ – 4.6 × 10^6^ CFU/mL) compared to untreated macrophages (5.5 × 10^6^ CFU/mL) (*p* < 0.05). These findings prompted us to elucidate the direct effect of MCL-1 on MAP survival in infected macrophages. The MCL-1 was inhibited pharmacology using Maritoclax (20–80 μM). As shown in Figure 5C, Maritoclax significantly decreased MAP survival in macrophages by 30%-38% compared to untreated macrophages (*p* < 0.05).

### 3.6. Notch Signaling Alters Apoptosis in MAP-Infected Macrophages

To evaluate the effect of infection on macrophage apoptosis, Caspase-3 activity was measured in THP-1-derived macrophages infected with MAP (10^7^ CFU/mL) for 24 h. MAP-infected macrophages resisted apoptosis with a slight decrease in Caspase-3 activity (7.3%), whereas heat-inactivated MAP and *M. smegmatis* showed 16.8% and 23.6% increase in Caspase-3 activity, respectively (*p* < 0.05). Then, to assess the role of Notch-1 signaling in regulating apoptosis in MAP-infected macrophages, Caspase-3 activity was measured in DAPT pre-treated THP-1-derived macrophages prior infection with MAP. Caspase-3 activity was elevated significantly in DAPT pre-treated macrophages by (20%–29.4%) compared to untreated macrophages (*p* < 0.05).

### 3.7. MAP Infection Modulates Macrophage Polarization

**Figure 6:**
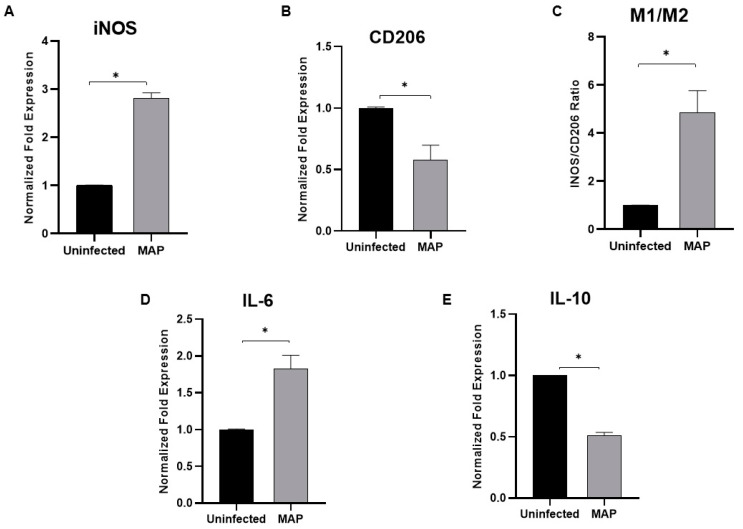
Effect of MAP infection on macrophage polarization and phenotypic response. THP-1-derived macrophages were infected with MAP (UCF 4, 10^7^ CFU/mL) for 24 h. Expression levels of iNOS (**A**), CD206 (**B**), IL-6 (**D**), and IL-10 (**E**) were measured using RT-PCR. M1/M2 ratio was determined by calculating the expression ratio of iNOS/CD206 (**C**). All experiments were performed in triplets. Data are shown as mean ± SD, and significance is considered as * *p* < 0.05.

The expression of iNOS as M1 pro-inflammatory macrophage marker and CD206 as M2 anti-inflammatory macrophage marker were examined following MAP infection (10^7^ CFU/mL) for 24 h. As shown in Figure 6, MAP infection caused upregulation of iNOS expression by 1.8-fold (A) and downregulation in CD206 by 0.42-fold (B) compared to uninfected cells (*p* < 0.05). M1/M2 ratio (C) was significantly high (3.8-fold) in MAP-infected cells compared to uninfected cells (*p* < 0.05). Furthermore, the expression of IL-6 and IL-10 were determined in MAP-infected macrophages. The results show a significant upregulation of IL-6 expression by ~1-fold (C) and a significant downregulation in IL-10 expression by 0.5-fold (D) compared to uninfected cells (*p* < 0.05).

### 3.8. Determination of Notch-1 Signaling Role in Macrophage Polarization in MAP-Infected Macrophages

**Figure 7:**
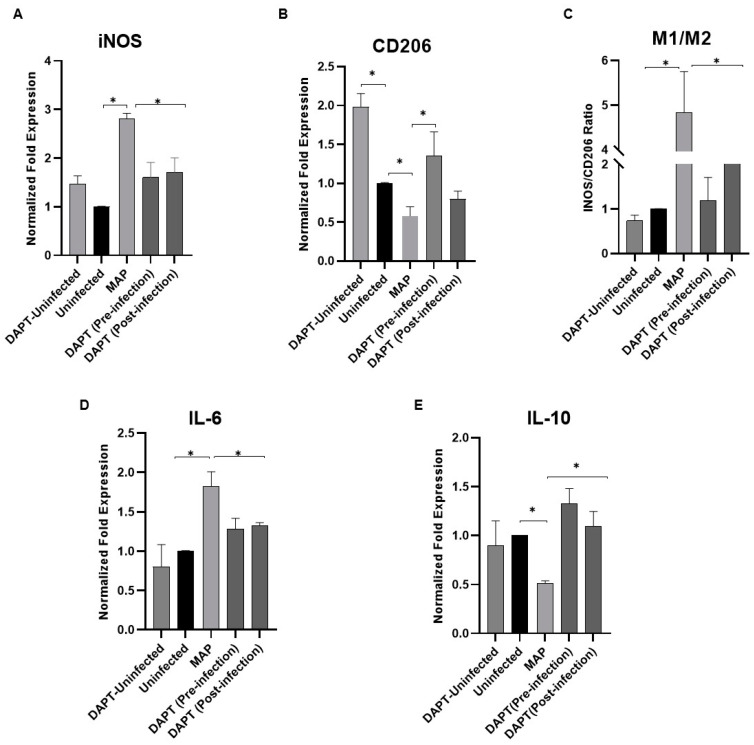
Notch-1 signaling modulates macrophage polarization in MAP-infected macrophages. Expression levels of iNOS (**A**), CD206 (**B**), IL-6 (**D**) and IL-10 (**E**) were measured using RT-PCR. M1/M2 ratio was determined by calculating the expression ratio of iNOS/CD206 (**C**). All experiments were performed in triplets. Data are shown as mean ± SD, and significance is considered as * *p* < 0.05.

Next, to explore the involvement of Notch signaling in macrophage polarization in MAP-infected macrophages, Notch signaling was blocked using DAPT (30 μM). Remarkably, treatment with DAPT prior to and post-infection diminished the MAP ability to induce iNOS expression by 1.2-fold, and 1.1-fold, respectively (*p* < 0.05). DAPT caused upregulation in CD206 expression (1.4 ± 0.3) in pre-infection treatment and (0.8 ± 0.1) in post-infection treatment compared to untreated infected cells (0.58 ± 0.1) (*p* < 0.05). The M1/M2 ratio significantly decreased in DAPT-treated cells prior infection (1.2 ± 0.52), and much more in post-infection (2.1 ± 0.52) compared to untreated infected cells (4.8 ± 0.9) (*p* < 0.05; Figure 7 A–C). Moreover, the expression of IL-6 was lower in DAPT-treated cells prior to infection (1.3 ± 0.13), and post-infection (1.3 ± 0.04) compared to MAP-untreated infected cells (1.8 ± 0.18) (*p* < 0.05). IL-10 expression was upregulated (1.3 ± 0.15) prior to infection, and (1.1 ± 0.15) in post-infection compared to untreated infected cells (0.51 ± 0.3) (*p* < 0.05; Figure 7D,E).

## 4. Discussion

The highly conserved Notch signaling has been accepted as an essential player in an immune system. The role of Notch signaling in lymphocyte activation and functions has been widely studied. However, its role in myeloid cells such as macrophages remains unclear [21]. This is despite the fact that several studies have hinted to a possible involvement of Notch signaling in macrophages during inflammation and infection [22,23]. Notch signaling appears to favor an inflammatory microenvironment in order to modulate the macrophage towards pro-inflammatory response [22,23]. For example, some studies reported a possible reciprocal modulation between Notch signaling and Toll-like receptor (TLR)-signaling and inflammatory cytokine signaling [23]. Fung et al. also reported the expression of specific Notch receptors and ligands on macrophages in response to pro-inflammatory and bacterial stimuli [22]. However, the specific role of Notch signaling in macrophages during bacterial infection is still unknown.

In general, *Mycobacteria* employ several survival tactics to escape macrophage-mediated elimination, such as switching the cell death type in infected macrophages from apoptosis to necrosis, which serves its survival and evades its antigen presentation to the adaptive immune system [10]. In this study, we demonstrated that Notch-1 signaling is involved in regulating macrophage apoptosis by inducing MCL-1 expression. MCL-1 is an anti-apoptotic protein that plays an essential role in macrophage viability and survival using several factors including STAT3 [14]. The latter is a transcription factor that is activated by various cytokines including IL-6 [15]. Previously, our group also reported that MAP induced IL-6 expression in infected macrophages model, which resulted in increased MAP survival [16]. We speculated then, that IL-6 expression may be directly regulated by Notch signaling which supports a report where a chromatin immunoprecipitation assay was used and detected the involvement of Notch-1 signaling in regulating IL-6 expression in an LPS-activated macrophage model [17]. Thereafter, we hypothesized that MAP infection may induce Notch-1 signaling and consequently increase IL-6 and MCL-1 expression and ultimately lead to a delayed macrophage apoptosis. The phenotypic outcome of this is an increase in bacterial persistence and overall inflammation. This study clearly demonstrated that Notch-1 was upregulated following MAP infection in THP-1-derived macrophages. The data in this study also indicate the MAP-induced Notch-1 signaling activation through the upregulation of HES-1 expression, a Notch target gene. The activation of Notch-1 signaling in MAP-infected macrophages was consistent with the upregulation of IL-6 and MCL-1. Our data confirm earlier observations in the macrophage model infected with *Mycobacterium Bovis*-BCG [24] and tuberculin-purified protein derivative (PPD) treatment [12]. Likewise, LPS treatment produced results similar to the MAP infection, which confirms the expected outcome when LPS is used [25]. As expected, our negative controls consisting of nonpathogenic *M. smegmatis* and heat-inactivated MAP were processed rapidly following phagocytosis and resulted in a minimum inflammatory response. This finding strongly associates MAP infection with Notch-1 signaling in modulating macrophage response and inflammation.

To further validate the role of Notch signaling in regulating IL-6 and MCL-1 expression, we blocked Notch signaling using the γ-secretase inhibitor known as DAPT. As expected, Notch-1 and HES-1 expression decreased significantly in infected and uninfected macrophages which supported our selection of the type of inhibitor used in this study. The impact of blocking Notch signaling was direct, causing a decrease in IL-6 and MCL-1 expression. Our data confirm earlier observations about reduced MCL-1 expression in another system using RAW264.7 cells and Notch-1 silencing technology [12]. Surprisingly, our data demonstrated a dramatic decrease in MCL-1 expression in response to DAPT treatment compared to Notch-1 and IL-6 response. This suggests a dual effect for Notch-1 and IL-6 in regulating MCL-1 expression. Most importantly, blocking Notch signaling with DAPT reduced MAP infection and the inflammatory response which strongly indicates that Notch signaling is essential for IL-6 and MCL-1 expression in response to intracellular infections such as MAP or *M. bovis.*

The essential role of IL-6 in promoting MAP survival in infected macrophages has been long studied in our lab [16], and we have always sought to unravel the underlying mechanism(s) involved in this observation. This study was designed to determine if Notch-1 may play a role here. Our data show a dramatic decrease in MAP viability in infected macrophages in response to DAPT treatment, and this finding directly links Notch-1 signaling to macrophage response in terms of defense mechanisms against MAP infection. The role of Notch signaling in the macrophage response was further confirmed following post-infection and pre-infection treatment with DAPT, which resulted in a decrease in MAP viability and inflammatory response which supports another report using lung mice infected with *M. tuberculosis* [25]. In contrast, the Notch inhibitor led to a higher virus load and inflammatory response in the lung mice during H1N1 virus infection [26]. To validate the intracellular effect of DAPT on Notch signaling and downstream expression, we determined the direct effect of this inhibitor on MAP in microbiologic culture media, and the result showed a partial reduction in extracellular MAP growth. DAPT is not an antibiotic and is not known to have antimicrobial activity. We speculate that the interference of DAPT in MAP culture growth may be related to post-translational modification such as protein glycosylation or lipoglycosylation; these processes include proteolysis via specific proteases and peptidases. Such modifications are essential for some enzymes and protein function and provide several physiological consequences in *Mycobacteria* [27]. In particular, we speculate that these proteolytic enzymes may be inhibited by proteolysis inhibitors such as γ-secretase inhibitors. This novel finding strongly suggests that targeting Notch signaling using DAPT will diminish MAP viability directly, as demonstrated extracellularly and indirectly through the potentiate macrophage response.

In various bacterial infection models, IL-6 was reported to induce DLL-1 expression in monocytes through the activation of STAT3. In turn, DLL-1 was noted to enhance IL-6 production and subsequent STAT3 activation [28]. This interplay between IL-6 and Notch ligand expression led us to extend our efforts to elucidate the possible interplay between IL-6 and Notch-1 signaling in macrophages. Our data revealed an induction in Notch-1 and HES-1 expression in THP-1-derived macrophages in response to exogenous rIL-6 treatment, which pinpoints the autocrine and paracrine effect of IL-6 on Notch-1 signaling and further emphasizes the positive feedback loop between IL-6 and Notch-1 signaling. Similarly, MCL-1 upregulation in response to rIL-6 treatment confirms the reciprocal modulation of IL-6 and Notch-1 signaling in regulating MCL-1 expression. This supports a previous report which suggested that STAT3 may play an essential role in inducing MCL-1 expression in macrophages [14]. Interestingly, targeting Notch signaling in rIL-6-treated macrophages weakened IL-6 to sustain MAP survival in infected macrophages, which confirms—one more time—the critical role of Notch signaling in MAP infection. To our surprise, blocking MCL-1 pharmacologically with Maritoclax caused a significant decrease in MAP viability and burden in infected macrophages. To our knowledge, this is the first time this novel finding is reported. While the data strongly support the role of Notch-1 in the modulation of macrophage response during infection, it also suggests that MCL-1 is involved and should be elucidated further.

Since *Mycobacteria* escape macrophage-mediated elimination by switching the cell death mode toward necrosis, and since MCL-1 inhibition decreases MAP survival in infected macrophages, we investigated if delayed apoptosis occurs during MAP infection. Our results revealed a decrease in Caspase-3 activity in MAP-infected macrophages compared to the uninfected group and macrophages exposed to heat inactivated MAP and non-pathogenic *M. smegmatis*. This is consistent with previous reports that virulent mycobacterial strains induce less apoptosis in THP-1-derived macrophages [29]. The decrease in Caspase-3 activity in MAP-infected macrophages is consistent with the upregulation of MCL-1 expression which suggests that MCL-1 is involved in inhibiting apoptosis during the MAP mode of action. We further confirmed this observation by targeting Notch signaling by DAPT which resulted in lessening the MAP ability to inhibit apoptosis. This novel finding links Notch-1 signaling in regulating macrophage response, apoptosis, and burden of MAP infection and exacerbating of the inflammatory response.

We further looked at a possible role for Notch signaling in macrophage polarization during MAP infection. As expected, and confirming what others reported in other microbial infections, we observed induction of M1 polarization *versus* M2 polarization in response to MAP infection and further confirmed it via the increased M1/M2 [30,31]. These data were validated by the elevated expression of IL-6 at the expense of IL-10 in these cells. Most importantly, blocking Notch signaling with DAPT during MAP infection reversed the M1/M2 ratio by switching the macrophage response towards an anti-inflammatory phenotypic response. This was further confirmed with the reduced IL-6 and increased IL-10 expression in these cells. The findings clearly support the critical role of Notch signaling in macrophage infection, especially in MAP-associated diseases, including CD and RA.

**Figure 8:**
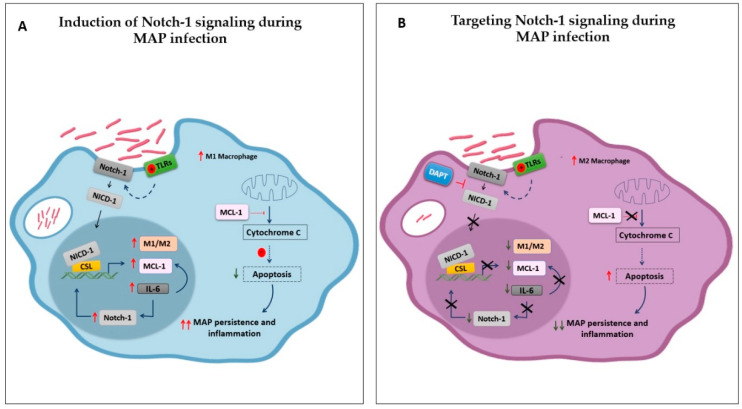
Schematic representation of the outcome of this study. (**A**): 1) MAP induces Notch-1 signaling and downstream influence on IL-6 to hijack MCL-1 dependent inhibition of apoptosis, which allow its chronic intracellular persistence, and subsequent inflammation. 2) Notch-1 and IL-6 reciprocal modulation to amplify MCL-1 expression, suggesting the critical role of Notch-1 signaling and its downstream effect in intensifying MAP-mediated effect in macrophages. 3) MAP promoting M1 versus M2 polarization through Notch-1 signaling. (**B**): Targeting Notch signaling in MAP-infected macrophages enhanced macrophage apoptosis, decreased MAP burden, and promoted M2 polarization and anti-inflammatory cytokines production. TLRs: Toll-like receptors, NICD: Notch intracellular domain.

Overall, this study provides new insight into molecular mechanisms involved in MAP-mediated infection in an in vitro macrophage system. The outcome clearly demonstrated that MAP activates Notch-1 signaling and has a downstream influence on IL-6 to hijack the MCL-1-dependent inhibition of apoptosis, which causes chronic intracellular persistence, and subsequent inflammation and possibly tissue damage. The study also provides data to support strategies based on the targeting of Notch signaling for therapeutic purposes (Figure 8). The crosstalk between Notch signaling and other pathways provides an opportunity for combinatorial treatment to target many pathways simultaneously, which may augment the therapeutic benefits.

## Figures and Tables

**Table 1:** Primer sequences.

Gene	Primer Sequence (5′-3′)	Amplicon Length (bp)
***β-actin***	CTCATCTTGTTTTCTGCGCAAGTT CTTCCCTCCTCAGATCATTGCTC	226
***Notch-1***	TGAAATTCAGGGCCCCTCC GCATCGGGCACCTGAAC	162
***HES-1***	CAGATCAATGCCATGACCTACCC GGAAAGCAAACTGGCCATCG	250
***MCL-1***	TGGTGGTGGTTGGTTAAAAGTCA GTGGAGTTCTTCCATGTAGAGGAC	152
***IL-6***	AGGAGAAGATTCCAAAGATGTAGCC TGCTCTAGAACCCAGCAAAGAC	228
***iNOS***	GGAGCAACGTTGAGGAAATAAGACT AAGAGCCAGAAGCGCTATCAC	252
***CD206***	GGAGGATTCCATGTATTTGTGAGC AAATGAGTGAAGTGAAATCAGTTACCT	510
***IL-10***	ATGTCTAGTTCAGGCAGTCCCA GGGCTTGCTCTTGCAAAACC	272
